# A pilot study: the development of a culturally tailored Malaysian Diabetes Education Module (MY-DEMO) based on the Health Belief Model

**DOI:** 10.1186/1472-6823-14-31

**Published:** 2014-04-08

**Authors:** Badariah Ahmad, Amutha Ramadas, Quek Kia Fatt, Anuar Zaini Md Zain

**Affiliations:** 1 Jeffrey Cheah School of Medicine and Health Sciences, Monash University Malaysia, Jalan Lagoon Selatan, Bandar Sunway, 47500 Petaling Jaya, Malaysia

**Keywords:** Self-efficacy, Validation, Education, Module, Knowledge, Health-belief model

## Abstract

**Background:**

Diabetes education and self-care remains the cornerstone of diabetes management. There are many structured diabetes modules available in the United Kingdom, Europe and United States of America. Contrastingly, few structured and validated diabetes modules are available in Malaysia. This pilot study aims to develop and validate diabetes education material suitable and tailored for a multicultural society like Malaysia.

**Methods:**

The theoretical framework of this module was founded from the Health Belief Model (HBM). The participants were assessed using 6-item pre- and post-test questionnaires that measured some of the known HBM constructs namely cues to action, perceived severity and perceived benefit. Data was analysed using PASW Statistics 18.0.

**Results:**

The pre- and post-test questionnaires were administered to 88 participants (31 males). In general, there was a significant increase in the total score in post-test (97.34 ± 6.13%) compared to pre-test (92.80 ± 12.83%) (p < 0.05) and a significant increase in excellent score (>85%) at post-test (84.1%) compared to pre-test (70.5%) (p < 0.05). There was an improvement in post-test score in 4 of 6 items tested. The remaining 2 items which measured the *perceived severity* and *cues to action* had poorer post-test score.

**Conclusions:**

The preliminary results from this pilot study suggest contextualised content material embedded within MY DEMO maybe suitable for integration with the existing diabetes education programmes. This was the first known validated diabetes education programme available in the Malay language.

## Background

Diabetes education and self-care in diabetes remains the cornerstone of diabetes management [1,2]. Norris *et al.* observed that diabetes self-management education (DSME) have evolved over the past four decades from primarily didactic interventions into the collaborative and theoretically based “empowerment” models [3].

In the United Kingdom, several structured diabetes education programmes were developed in an effort to provide better diabetes care. For instance, diabetes education of self-management for on-going and newly diagnosed (DESMOND) type 2 diabetes patients [4] and expert patient education versus routine treatment (X-PERT) [5] and dose adjustment for normal eating (DAFNE) for type 1 diabetes patients [6] were developed and implemented. Contrastingly, evidences of theoretically based diabetes education programmes are scanty in Malaysia.

Due to explosion of diabetes in Malaysia [7-10], an executive decision made by various policymakers to increased resource centres (e.g. one-stop diabetes centre) in primary care settings and hospitals to improve diabetes care delivery [11]. Worryingly, despite the multiple-pronged efforts, glycaemic control in Malaysia remained suboptimal [12-19].

The aim of this pilot study was to review existing diabetes education programmes both in Malaysia and abroad and consequently develop a culturally suitable diabetes education module based on the Health Belief Model.

## Methods

### Needs assessment for a contextualised Malaysian Diabetes Education Module (MY DEMO) in Malay

Evidence on structured diabetes education study in Malaysia has been scarce. A recent study by Tan *et al.* using a structured education programme based on self-efficacy theoretical framework succeeded in short term improvement of glycated haemoglobin, three self-care practices and diabetes knowledge in a cohort of poorly controlled diabetes patients [20].

**Figure 1:**
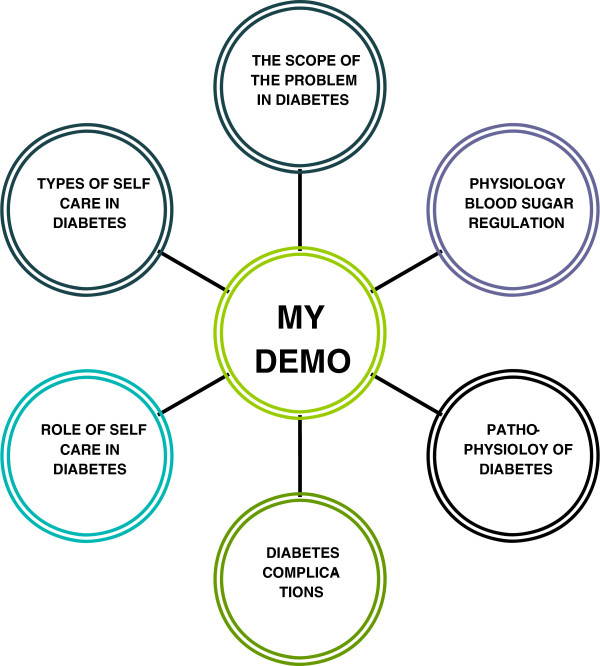
The important aspects highlighted in the MY DEMO.

The American Association of Diabetes Educators (AADE) defined the seven essential self-care behaviours for successful and effective diabetes self-management [21]. These 7 self-care behaviours (AADE7™) include healthy eating, being active, monitoring, taking medication, problem solving, healthy coping and reducing risks. Majority of these essential self-care components were embedded within MY DEMO (Figure 1).

Recent evidences had shown that culturally tailored packages were useful in helping diabetes patients in managing their diabetes. These packages included information that was easy to convey, with appealing visual teaching tools and that were interactive and effective in delivering diabetes education to high risks indigenous groups [22,23]. Vincent *et al.* opined that specific cultural modifications such as incorporation of dietary preferences and cultural beliefs, delivery in mother tongue language and using bilingual nurses and dieticians can enhanced diabetes self-management and result in improved glycaemic control [24].

### Theoretical background

While it is well known that knowledge alone did not always predict behaviour change or glycaemic control [25], health behaviour theories would generally include basic knowledge as a necessary element of health behaviour change and outcomes [26]. Many health models developed in the past five decades had tried to describe, predict, explain and ultimately change health behaviours. Most of the well-known health behaviour models have their roots in the field of psychology; Health Belief Model (HBM) by Rosenstock [27]; Social Cognitive Theory (SCT) by Bandura [28]; Transtheoretical Model (TTM) by Prochaska and DiClemente [29] and more recently Health Action Process Approach (HAPA) by Schwarzer [30]. MY DEMO was developed based on HBM and made clear attempts to measure some of the constructs such as cues to action, perceived benefit and perceived severity. In addition, the other three constructs such as perceived susceptibility, perceived barriers and self-efficacy were integrated into MY DEMO.

### Development of MY DEMO

#### Culturally tailored components in MY DEMO development

Malaysia is a multicultural country and the Malays, Chinese and Indians form the fabric of the society. Although *Malay* language is widely spoken, Mandarin and Tamil are frequently used too. The first key concept the author wanted to highlight was the common misnomer used to describe diabetes. It is well known that diabetes is due to the dysfunctional sugar metabolism due to the lack of insulin and/or insulin resistance leading to hyperglycaemia. There was an emphasis to educate participants on the importance of the term “sweet blood” (*darah manis* in Malay) rather than “sweet urine”. This was to make participants aware that the issue in diabetes is hyperglycaemia and not glycosuria - which usually presents later when the diabetes complications had ensued. The terminology “sweet urine” is a common reference for diabetes patients and general population in Malaysia (i.e. *kencing manis* in *Malay*, *inippu neer* in *Tamil* and *tang-niow* in *Mandarin*), as most people associate the diabetes condition with glycosuria and not hyperglycaemia. Glycosuria (*kencing manis*) was highlighted as a side-effect of a declining kidney function and hence the importance of early detection of hyperglycaemia (*darah manis*) in diabetes diagnosis. Other core topics such as patho-physiology of diabetes, the recognition of diabetes symptoms and signs, complications and prevention of diabetes were also emphasized using simple *Malay* language. Visual and colorful diagrams were employed to illustrate the complex and essential concepts which can lead to insulin resistance.

Simple analogies were also used to enhance understanding of the role of insulin by explaining how the insulin “key” can open the cell “door” and help normalize blood sugar level in the body. In addition, the authors likened the “national road transport system” as an equivalent to a vascular system in the body which can be damaged due to prolonged and uncontrolled hyperglycaemia. The diabetes complications such macro-vascular and micro-vascular diseases were clearly illustrated using visual aids such photographs, anatomical diagrams and animations. The HBM constructs such as perceived susceptibility, perceived severity, perceived benefits, perceived barriers, cues to action and self-efficacy were embedded in the module during the development of MY DEMO. Finally, other key messages emphasised in MY DEMO were healthy lifestyle modifications including balance diet and regular exercise. Popular local physical activities (i.e. *tai chi, qi gong, yoga,* brisk walking) were also mentioned to incorporate active lifestyle into daily activities.

#### Delivery of MY DEMO

Every effort was made to ensure that content could be easily followed by participants. Some of the key elements of plain language defined by Kandula *et al.* are; a) delivering important information first b) breaking complex information into understandable chunks c) using simple language and d) defining technical terms were adopted during MY DEMO delivery [31]. The delivery of MY DEMO was through a one-hour didactic lecture followed by another hour of dialogue session to the group participants. In total, MY DEMO talk was delivered thrice by the same author (BA). Different types of format were used during MY DEMO delivery to address different types of learning styles that may be employed by participants [32,33]. For example, the author used visual illustrations, audio animations and peer discussion to engage the participants during the session. Later, a group discussion ensued to address any other questions or issues related to the diabetes lecture.

### Research tools

#### Development of pre- and post-questionnaires for the diabetes module

The module was evaluated in this pilot study using pre- and post-test questionnaire. The initial step of item generation garnered a large pool of questions (18 items and 37 sub-items). Consequently, all the items were examined for face and content validity by a panel of experts and non-experts from the faculty. The panellists consisted of two endocrinologists, two general practitioners and two basic scientists who are fluent in both the English and *Malay* language. Based on their expertise and background knowledge on diabetes, the panellists were asked to first scan through the questions for face validity. Consequently, the panellist were asked to rank the questions according to the level of difficulty by the help of a Likert-scale of easy, moderate, difficult or not suitable as part of the content validity exercise.

Generally the questions were divided into sections; (a) general diabetes information (eg. signs and symptoms, risk factors, pathophysiology, diabetes complications, hypoglycaemia) and (b) self-care practices (healthy eating, exercise, foot care, self-monitoring blood glucose). Briefly, easy-type questions consisted of basic information about pathophysiology of diabetes (i.e. insulin hormone deficiency) and common diabetes complications (i.e. blindness and kidney failure). Examples of moderate-type questions are like benefits of exercise on blood sugar and cholesterol, types of food that convert to glucose and normal level of fasting and 2 hours post prandial sugars. Difficult-type questions tested complex issues like pathophysiology of diabetes, insulin resistance and lack of glucose absorption. In addition, the panellists also commented on the suitability of the language and ensured that the questions phrased were jargon-free and using simple *Malay* language. Items deemed unsuitable were removed from the pool of questions. Finally, six items (2 ranked easy; 3 ranked moderate; 1 ranked difficult) were selected and used in the pre- and post-test questionnaires (Table 1).

**Table 1:** Measurement of Health Belief Model constructs and ranking system for the 6 items used in pre- and post-test questionnaire

**Section**	**Questions 3 options (1 correct answer)**	**Item code**	**Difficulty index**	**Construct**
	What types of food will be converted to glucose once it has been digested?	Q1	Easy	Cues to action
	***● Carbohydrate***			
	***●*** *Vitamin*			
	***●*** *Fat*			
	After eating a meal, what hormone is responsible to reduce blood sugar level?	Q3	Moderate	Cues to action
	***● Insulin***			
	***●*** *Glucagon*			
	***●*** *Adrenaline*			
	In diabetes, “sweet blood” or hyperglycaemia can affect all the blood vessels in the body. Which is a common diabetes complication?	Q4	Moderate	Perceived severity
	***● Kidney dialysis***			
	***●*** *Liver transplant*			
	***●*** *Knee transplant*			
	What factor leads to “sweet blood” in diabetes patients?	Difficult		Cues to action
	***●*** *Fat cells able to absorb sugar*			
	***●*** *Muscle tissues able to absorb sugar*			
	***● Pancreas gland produce less insulin***			
	Foot-care is important in diabetes. What should you do to look after your feet?	Q2	Easy	Perceived benefit
	***●*** *Do not wear any shoes when walking outdoors*			
	***● Keep the spaces between your toes dry***			
	***●*** *Treat any foot ulcer or wound by yourself*			
	Benefits of exercise	Q5	Moderate	Perceived benefit
	***● Increase insulin sensitivity***			
	***●*** *Increase blood sugar*			
	***●*** *Increase blood pressure*			

Briefly, four items tested the general knowledge of diabetes section (Q1, Q3, Q4 and Q6) and two items tested the self-care practices section (Q2 and Q5). All the HBM constructs embedded within these items and were measured subsequently in the pre- and post-questionnaires. Participants had five to ten minutes to read and complete the pre and post questionnaires before submitting them. Each question had three options and only one correct answer.

The participants had to answer all questions. Failure to select any one or selecting more than one option was considered incorrect and no marks given. The score is categorised as the following: 74.99% or below as failure to understand the content, 75–79.99% as pass, 80–84.99% as good and ≥85% of above as excellent.

#### Evaluation tool and process

The participants were asked to evaluate the objectives and content material of MY DEMO (e.g. physiology of body, definition, signs and symptoms, complications and prevention of diabetes and identification of hypoglycaemia symptoms). A 4-item Likert-scale was developed for participants to select if the items (mentioned above) were (i) very useful (ii) useful (iii) less useful and (iv) not at all useful.

### Study sample

The sample size calculation, which was carried out using GPower software [34] was based on improvement in diabetes knowledge score in the intervention group as reported by Vincent et al. [24]. From the literature review, the baseline value for mean diabetes knowledge score was 15.11 with standard deviation (sd) 2.6. Post intervention has shown that the mean diabetes knowledge score has increased to 16.89 with sd 3.3. A sample size of 72 was required to detect this mean and sd difference at significance (α) 0.05 and power (1-β) = 0.80 (80%).

Convenience sampling was used in this pilot study from two government organisations and a private industrial sector in three states (Selangor, Kuala Lumpur and Johor) in Peninsular Malaysia. Two weeks prior to the arranged date, all potential participants received an in-house e-mail to inform them about the MY DEMO talk from their respective organisations. An in-house e-mail was administered to increase the dissemination of news regarding the scheduled talk. In addition, a reminder e-mail was circulated a few days before the talk to ensure as many staff could attend the talk. Of note, the author could only get verbal feedback from the respective human resource departments regarding the number of interested parties who might be coming on the scheduled day. Nevertheless, some participants attended the talk on the day itself after hearing from their work colleagues and others could not attend due to competing work commitments. Consequently following the recruitment exercise, ninety-one Malaysian adults (18 years and above) with minimum secondary (O-Level equivalent) level education and conversant in the *Malay* language, were recruited to evaluate the diabetes education package. The participants were predominantly Malays (96.6%). All participants gave their consent to attend the module and agreed to complete the pre – and post-test questionnaires. This study received ethics approval from the Ministry of Health, Malaysia.

### Statistical analysis

Mean and standard deviation were calculated for continuous variables, while frequency tables were constructed for categorical variables. Paired *t*-test was used to determine the mean differences of scores, while chi-square or Fisher’s Exact test was used to determine the association between categorical variables. All statistical analyses were performed with IBM® PASW® Statistics 17.0. The significance level was set at p = 0.05.

## Results

### Comparison of pre and post-test results

Eighty-eight participants (response rate = 96.7%) completed both pre- and post-test questionnaires, while all participants (n = 91) completed the process evaluation questionnaire. Table 2 presents the responses given by the participants to all the items. There was an increase in correct answers to all items related to perceived benefits, and two items related to cues to action. The increase in number of correct answers for item Q1 at post-test was significant (p < 0.01). There is a slight decrease in correct answers to item Q4, which measured the perceived severity. Item Q6, which was ranked as difficult, had the lowest number of correct answers in post-test (93.2%).

**Table 2:** Correct responses at pre- and post-test

**Construct (HBM)**	**Item’s code**		**Pre-test**	**Post-test**	**P value**
**Perceived benefit**	Q5	Correct	83 (94.3)	88 (100.0)	n/a
**Perceived benefit**	Q2	Correct	71 (80.7)	85 (96.6)	0.479
**Cues to action**	Q1	Correct	84 (95.5)	86 (97.7)	0.002*
	Q3	Correct	88 (100.0)	87 (98.9)	n/a
	Q6	Correct	76 (86.4)	82 (93.2)	0.188
**Perceived severity**	Q4	Correct	87 (98.9)	86 (97.7)	1.000
**Total score**		Mean ± SD	92.80 ± 12.83	97.35 ± 6.13	0.004*
**Category of score, n (%)**		Excellent (>85%)	62 (70.5)	74 (84.1)	0.005*
		Good (80–84.99%)	17 (19.3)	14 (15.9)	
		Pass (75 – 79.99%)	9 (10.2)	0 (0.0)	

*Significant at p < 0.01.

Results in Table 2 also presents the differences between pre- and post-test scores. There was a significant increase in the total score in post-test (97.34 ± 6.13%) compared to pre-test (92.80 ± 12.83%) (p < 0.01). There was an increase in number of participants who obtained excellent score (>85%) at post-test (84.1%) compared to pre-test (70.5%), while there were decreases in those scoring fair (50–69.99%) and good (70–84.99%). Notably, there were no very poor or poor scores in both the pre-test and post-test questionnaire.

## Discussion

### Implications of MY DEMO results

The items selected in the pre and post-test questionnaire were to emphasised important issues regarding the application of diabetes knowledge in optimising care in diabetes patients. Although only some of the HBM constructs were selected in the pilot project, in future intervention study, other constructs such as perceived susceptibility, perceived barriers and self-efficacy will be considered too [27].

In the questionnaire, the authors chose to highlight three items related to cues to action in order to provide “how-to” or applied information and promote awareness about diabetes. For example, item Q1 (cues to action) pertaining types of food eaten was considered as basic knowledge. There was no issue with this item and all participants scored significantly higher post-test. The significantly higher score amongst the excellent category also confirmed majority of participants benefited from MY DEMO.

Contrastingly, the results of item Q3 pertaining role of insulin hormone and item Q6 pertaining pathophysiology of diabetes showed a decline in the score. The reported knowledge gap in these two areas should be given due consideration by the authors when implementing future intervention study. In addition, the low score for this item (Q6) was somewhat expected as this item was ranked as a “difficult” question by the panel of experts during item generation. However, as part of an assessment mechanism of MY DEMO, the authors had decided to include at least one “difficult” question in each assessment item module.

Item Q4 measured perceived severity showed a reduction in post–test score. The aim of item Q4 was to prompt patients *vis-a-vis* the pitfall of sustained “sweet blood” and the damage it can cause to the circulatory system leading to micro- and macro-vascular damage. Again, this negative finding is relevant to the authors as it identified some of the gaps in participant’s understanding of diabetes complications and should be address when delivering MY DEMO in future intervention study.

There were no difficulties with the two items (Q2 and Q5) which measured perceived benefits of diabetes self-care practices such as foot care and exercise. Item Q2 that emphasised on foot care was selected as it defined simple actions that can be taken by patients in order to prevent common diabetes complications such as infection and amputation. Furthermore, item Q5 focused on the benefits of physical activity on the body’s glucose metabolism and blood pressure control, which is essential in optimising diabetes control. Nevertheless, the authors opined that these items are important aspects of diabetes management and should be reinforced in the module and tested in the questionnaire to ensure participants understanding.

The authors also acknowledged a small discrepancy between those who completed the evaluation questionnaires (n = 91) compared to those who completed the pre- and post-test questionnaires (n = 88). This small discrepancy (3.3%) occurred because three of the participants did not complete the post-test questionnaires. Although the authors tried to make contact with the participants, *via* the Human Resource departments, the effort was not fruitful and the questionnaires remain incomplete. However, given the high response rate (96.7%) the authors posit that this small discrepancy is not significant.

### Inconsistent health outcomes from past diabetes education programme studies

Previous literature review on diabetes self-management education has found short-term (<6 months) positive effects on knowledge, dietary habits and glycaemic control. A meta-analysis has shown a decrease in HbA1C of 0.8% at immediate follow up and 0.3% at 4 months or longer follow-up. Hence, the benefit of self-management education on glycated haemoglobin had been shown to decrease between 1 and 3 months and not sustained for long term [35]. However, the recent X-PERT programme succeeded in showing sustained improvement for glycaemic control and other secondary outcomes such as body weight, BMI and waist circumference, reduction of diabetes medication, knowledge of diabetes, self-empowerment and self-management skills at 14 months [5]. Remarkably, although, the DESMOND study succeeded in showing improvement in weight loss, smoking cessation and positive improvement in beliefs about illness at one year it did not show sustained improvement in glycated haemoglobin [4].

A further 3-year post intervention DESMOND study also failed to show sustained improvement in biomedical and lifestyles outcomes, although some changes to illness beliefs were sustained [36]. Contrastingly, a long-term Italian study - rethink organisation to improve education and outcomes (ROMEO) had shown favourable clinical, cognitive and psychological outcomes following a 4-year study of a continuous diabetes education programme [37].

### Other potential primary outcomes to measure a diabetes education programme

There had been some criticisms about the limited short-term improvement in glycaemic outcome in many diabetes education programmes [4,6]. Hence the role of glycated haemoglobin as the primary outcome to measure the effectiveness of a diabetes education intervention programme have been questioned. Cooper *et al.* argued there were other issues that may also be important to the diabetes patients. He posited the success of diabetes education should be regarded as having wide benefits, and should not be judged only by standard clinically based endpoints [38].

The authors are aware of some of these challenges and must identify the gaps early on in the development and implementation of a diabetes education programme. Hence, a point of consideration in our future intervention study is to adopt a multi-approach intervention like the use of teaching videos and regular telephone consultations. The authors will also include other primary outcomes such as self-efficacy, compliance and motivation in addition to glycated haemoglobin in an attempt to measure the wider benefit of the effectiveness of the programme.

### Strengths and limitations

To our knowledge, ours was the first study to validate a diabetes education module in the *Malay* language. The content of MY DEMO was contextualised to emphasis important messages and hopefully facilitates transfer of knowledge by using familiar keywords, common analogies and concepts. The delivery of MY DEMO was kept standardised with minimal variation, as the main author was also the sole presenter during these sessions.

However, the authors would like to highlight the limitation of convenience sampling used in this pilot study. Firstly, participants were from the public rather than known diabetes patients. This decision was partly because of the ease of delivering MY DEMO in a timely manner. However, demographic characteristics of the participants showed majority were Malays and conversant in the *Malay* language and the sample is representative of future cohort of patients. Thirdly, participants must have at least secondary level education in order to follow the basic content of MY DEMO. The same inclusion criteria of literacy will be built-in for future intervention study. Hence, it is reasonable to surmise the results of this pilot study can be generalised with the cohort of diabetes patients in future intervention study.

## Conclusions

The results of the pilot study suggest that MY DEMO would be suitable as part of a diabetes management strategy particularly in the aspect of patient education. This was the first known diabetes education programme in the *Malay* language that had attempted to use the health belief model as its framework and measure its outcomes using the known constructs. The preliminary result is positive and suggests that MY DEMO might be beneficial as part of or in addition to the existing diabetes education programme.

## Abbreviations

MY DEMO: Malaysian Diabetes Education Module; HBM: Health-belief Model; DESMOND: Diabetes Education of Self-Management for ON-going and Newly Diagnosed (for type 2 diabetes patients); DAFNE: Dose adjustment for normal eating (for type 1 diabetes patients); X-PERT: EXpert Patient Education versus Routine Treatment; AADE: American Association of Diabetes Educators; ROMEO: Rethink Organisation to improve Education and Outcomes.

## Competing interest

The authors declare that they have no competing interests.

## Authors’ contribution

BA is the main author of the research paper. She has made substantial contributions to development and validation of the diabetes education module (MY DEMO), conception and design of the study and acquisition, analysis and interpretation of data. AR has contributed towards the validation of the research tools (e.g. questionnaires), analysis and interpretation of data. QKF has contributed in the design of the study, determination of sample size and validation of the research tools (e.g. questionnaires), analysis and interpretation of data. AZ has contributed in the overall concept of the study design, development of the diabetes education module and interpretation of data. All authors read and approved the final manuscript.

## Authors’ information

BA is a Senior Lecturer and currently pursuing her PhD is Diabetes Education. Her doctoral topic is looking at the impact of education and self-management skills in optimising diabetes management.

AR is a Lecturer and recently completed her doctoral in Public Health. She is a trained nutritionist and clinical researcher.

QKF is an Associate Professor in Community Health and Medical Statistician.

AZ is a Professor of Endocrinology axnd the Head of the Jeffrey Cheah School of Medicine and Health Sciences, Sunway campus.

## Pre-publication history

The pre-publication history for this paper can be accessed here:

http://www.biomedcentral.com/1472-6823/14/31/prepub
